# 

**Published:** 1863-11

**Authors:** 


New Series, Vol. 6, No. 11. Whole Series, Vol. 20, No. 11.

Box 1305

### THE CHICAGO

### MEDICAL JOURNAL.

DANIEL BRAINARD, M. D.,
J. ADAMS ALLEN, M. D.,
Editors and Proprietors.

NOVEMBER, 1863.

CHICAGO:

J. C. W. BAILEY, BOOK AND JOB PRINTER, 130 CLARK STREET.

1863.

